# Supplementary material for: Drug repurposing for aging research using model organisms
Source: Aging Cell. 2017 Jun 16;16(5):1006–15. doi: 10.1111/acel.12626 (PMC5595691; doi:10.1111/acel.12626)
Supplement: Supplementary file 7 — Data S1 Zip‐Archive of all report cards. [file ACEL-16-1006-s007.zip › RC_PGH.pdf]

PGH

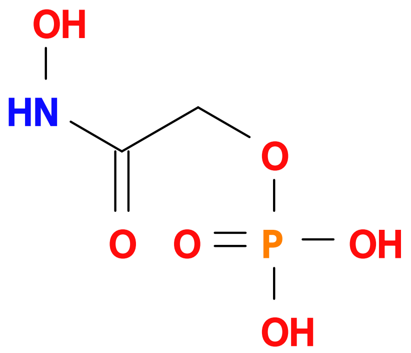

Database identifiers

ChEMBLCompound	CHEMBL371668
DrugBank	DB03026
CHEBI	28475

Ranking

	Rank	Score
Drosophila	217/697	0.68
C. elegans	218/591	0.236

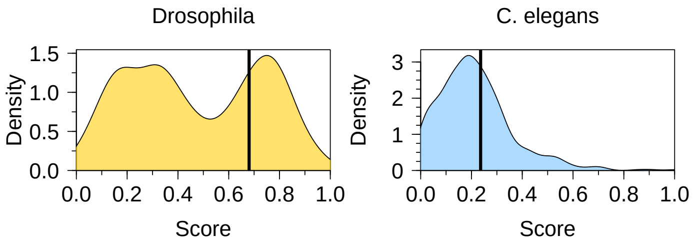

	Ageing implication	Domain conservation	Binding site conservation	Binding affinity	Bioavailability	Lipinski	Promiscuity	Purchasability	Drug approval	**Total**
Drosophila	1.0	0.921	0.981	0.745	(0.9)	0.0	-0.0	0.0	0.075	0.68
C. elegans	1.0	0.914	0.981	0.745	0.242	0.0	-0.0	0.0	0.075	0.236

Names

- **phosphoglycolohydroxamic acid**
- Phosphoglycolohydroxamate
- Phosphoric Acid Monohydroxycarbamoylmethyl Ester

Roles

ChEBI entry 28475 has no roles

Status

Approved drug (according to ChEMBL)	No
Classification (according to DrugBank)	experimental
Number of Rule of 5 violations	0
Binding affinity to original target in log units (RF-Score prediction)	6.07
Burns *C. elegans* bioavailability prediction	-2.52

Compound Target Characteristics

Triosephosphate isomerase

Best gene implication in ageing for this target family came from gene P29613 annotated in UniProt release 2014_02. Annotation GO 8340 (determination of adult lifespan) was Inferred from Mutant Phenotype

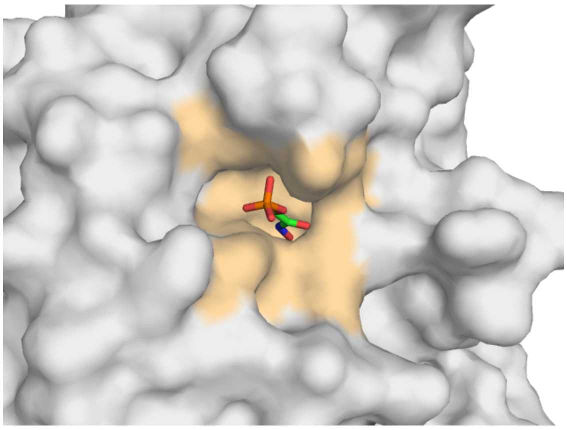

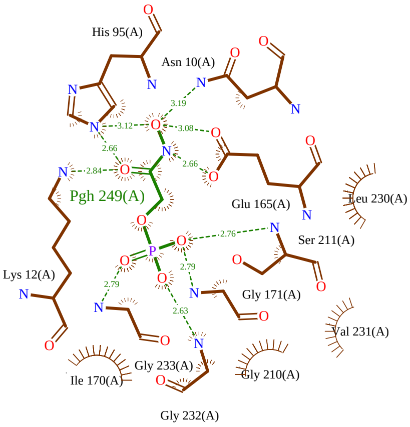

protein	amino acids contacts (binding site)
PDB:3ypi:chainA:P00942	N K Q E A I G G S L V G G
PDB:7tim:chainA:P00942	N K H E A I G G S L V G G
sp:P60174:TPIS_HUMAN	N K H E A I G G S L V G G
tr:M0R4U4:M0R4U4_RAT	N K H E A I G G S L V G G
sp:P48500:TPIS_RAT	N K H E A I G G S L V G G
sp:P17751:TPIS_MOUSE	N K H E A I G G S L V G G
sp:P29613:TPIS_DROME	N K H E A I G G S L V G G
sp:Q10657:TPIS_CAEEL	N K H E A I G G S L V G G
sp:P00942:TPIS_YEAST	N K H E A I G G S L V G G

	whole protein		domain-based		contact-based	
protein	ident	simil	ident	simil	ident	simil
PDB:3ypi:chainA:P00942	1.0	1.0	1.0	1.0	1.0	1.0
PDB:7tim:chainA:P00942	1.0	1.0	1.0	1.0	0.92	0.98
sp:P60174:TPIS_HUMAN	0.46	0.72	0.53	0.84	0.92	0.98
tr:M0R4U4:M0R4U4_RAT	0.46	0.75	0.49	0.79	0.92	0.98
sp:P48500:TPIS_RAT	0.51	0.82	0.52	0.83	0.92	0.98
sp:P17751:TPIS_MOUSE	0.43	0.69	0.52	0.83	0.92	0.98
sp:P29613:TPIS_DROME	0.56	0.85	0.56	0.85	0.92	0.98
sp:Q10657:TPIS_CAEEL	0.54	0.84	0.54	0.84	0.92	0.98
sp:P00942:TPIS_YEAST	1.0	1.0	1.0	1.0	0.92	0.98

Tpi (FBgn0086355) associated phenotypes

bang sensitive, chemical conditional, chemical resistant, chemical sensitive, fast, heat sensitive, heat stress response defective, neuroanatomy defective, oxidative stress response defective, short lived, stress response defective

(Information from FlyBase)

Tpi (UniProt:P29613) annotation
Pathway: Carbohydrate biosynthesis; gluconeogenesis.
Pathway: Carbohydrate degradation; glycolysis; D-glyceraldehyde 3- phosphate from glycerone phosphate: step 1/1.
Subunit: Homodimer.
Developmental stage: Present in substantial amounts in oocytes, declines in abundance in early embryos, and begins to increase during mid-embryogenesis. Levels peak in the third instar larva then decline during pupal stages, rising again near the time of eclosion. (PubMed:1720860).

(Information from UniProt)

tpi-1 (UniProt:Q10657) annotation
Pathway: Carbohydrate biosynthesis; gluconeogenesis.
Pathway: Carbohydrate degradation; glycolysis; D-glyceraldehyde 3- phosphate from glycerone phosphate: step 1/1.
Subunit: Homodimer.

(Information from UniProt)
